# Serial CT analysis in idiopathic pulmonary fibrosis: comparison of visual features that determine patient outcome

**DOI:** 10.1136/thoraxjnl-2019-213865

**Published:** 2020-04-28

**Authors:** Joseph Jacob, Leon Aksman, Nesrin Mogulkoc, Alex J Procter, Bahareh Gholipour, Gary Cross, Joseph Barnett, Christopher J Brereton, Mark G Jones, Coline H van Moorsel, Wouter van Es, Frouke van Beek, Marcel Veltkamp, Sujal R Desai, Eoin Judge, Teresa Burd, Maria Kokosi, Recep Savas, Selen Bayraktaroglu, Andre Altmann, Athol U Wells

**Affiliations:** 1 Department of Respiratory Medicine, University College London, London, UK; 2 Centre for Medical Image Computing, University College London, London, UK; 3 Department of Respiratory Medicine, Ege University Hospital, Izmir, Turkey; 4 Department of Radiology, University College London Hospitals NHS Foundation Trust, London, UK; 5 Department of Radiology, Royal Free Hospital, London, UK; 6 Department of Radiology, Royal Brompton and Harefield NHS Foundation Trust, London, UK; 7 NIHR Biomedical Research Centre and Clinical and Experimental Sciences, University of Southampton, Southampton, Hampshire, UK; 8 Department of Pulmonology, St Antonius Hospital, Utrecht, The Netherlands; 9 Division of Heart and Lungs, University Medical Center Utrecht, Nieuwegein, Utrecht, The Netherlands; 10 Department of Respiratory Medicine, Aintree University Hospitals NHS Foundation Trust, Liverpool, UK; 11 Department of Radiology, St. George's Hospital, London, Greater London, UK; 12 Interstitial Lung Disease Unit, Department of Respiratory Medicine, Royal Brompton and Harefield NHS Foundation Trust, London, UK; 13 Department of Radiology, Ege University Hospital, Izmir, Turkey

**Keywords:** idiopathic pulmonary fibrosis, bronchiectasis, imaging/CT

## Abstract

**Aims:**

Patients with idiopathic pulmonary fibrosis (IPF) receiving antifibrotic medication and patients with non-IPF fibrosing lung disease often demonstrate rates of annualised forced vital capacity (FVC) decline within the range of measurement variation (5.0%–9.9%). We examined whether change in visual CT variables could help confirm whether marginal FVC declines represented genuine clinical deterioration rather than measurement noise.

**Methods:**

In two IPF cohorts (cohort 1: n=103, cohort 2: n=108), separate pairs of radiologists scored paired volumetric CTs (acquired between 6 and 24 months from baseline). Change in interstitial lung disease, honeycombing, reticulation, ground-glass opacity extents and traction bronchiectasis severity was evaluated using a 5-point scale, with mortality prediction analysed using univariable and multivariable Cox regression analyses. Both IPF populations were then combined to determine whether change in CT variables could predict mortality in patients with marginal FVC declines.

**Results:**

On univariate analysis, change in all CT variables except ground-glass opacity predicted mortality in both cohorts. On multivariate analysis adjusted for patient age, gender, antifibrotic use and baseline disease severity (diffusing capacity for carbon monoxide), change in traction bronchiectasis severity predicted mortality independent of FVC decline. Change in traction bronchiectasis severity demonstrated good interobserver agreement among both scorer pairs. Across all study patients with marginal FVC declines, change in traction bronchiectasis severity independently predicted mortality and identified more patients with deterioration than change in honeycombing extent.

**Conclusions:**

Change in traction bronchiectasis severity is a measure of disease progression that could be used to help resolve the clinical importance of marginal FVC declines.

**Key messages**

**What is the key question?**

- In patients with idiopathic pulmonary fibrosis (IPF) with marginal annualised forced vital capacity (FVC) declines (5.0%–9.9%), can visual evaluation of serial CT scans distinguish genuine clinical deterioration from measurement inaccuracy?

**What is the bottom line?**

- Change in traction bronchiectasis independently predicts mortality in patients with IPF with marginal FVC declines, identifies more patients with disease worsening than honeycombing change and is associated with good interobserver agreement.

**Why read on?**

- Annualised rates of FVC decline that lie within the range of measurement noise are increasingly common in patients with IPF receiving antifibrotics, necessitating simple accessible methods to determine whether marginal FVC declines truly reflect patient deterioration for both clinical management and future non-inferiority drug trials.

## Introduction

The introduction of antifibrotic medication for the treatment of IPF has resulted in a reduction in the rate of forced vital capacity (FVC) decline.^1 2^ Fewer patients will now undergo definitive declines of ≥10% of FVC, and more patients will be seen with declines that lie within the range of measurement variation (between 5.0% and 9.9% annualised FVC declines). There are also trials under way examining the prognostic benefit of antifibrotic medication in non-IPF fibrosing lung diseases^3 4^ where patients are likely to undergo less dramatic declines than are seen in IPF. Knowing whether marginal declines in FVC values reflect measurement variability or genuine clinical deterioration is therefore going to be an increasingly challenging problem both in clinical practice and in future drug trials in fibrosing lung diseases.

CT analysis has been considered as a complementary tool to FVC measurement, whereby identification of worsening of disease on CT could be used to confirm that a marginal FVC decline reflects clinical deterioration. To date, however, the focus has been on quantitative CT analysis,^5^ which can be expensive and of limited availability. Our study chose to examine whether change in visual CT parameters could be used in the same way as quantitative tools in adjudicating marginal FVC declines. We examined CT parameters routinely evaluated by radiologists and examined change on a simple 5-point scale. As well as global measures of CT pattern change, we examined whether lobar scores of change in interstitial lung disease (ILD) extent added more prognostic information than global ILD change measures.

## Methods

### Study population

Cohort 1 consisted of patients diagnosed by a multidisciplinary team with IPF according to published guidelines,^6^ presenting to the Royal Brompton Hospital, London, with longitudinal CT imaging performed between 2007 and 2014. Cohort 2 comprised patients presenting to St. Antonius Hospital, Utrecht (between 2004 and 2015), Ege Hospital Izmir, Turkey (between 2008 and 2015), and Southampton General Hospital, UK (between 2013 and 2015). Patients were included in the study if they had undergone two non-contrast, supine, volumetric thin section CT scans (maximum collimation of 2 mm) within a time period of 6–24 months. Pulmonary function tests (PFTs) evaluated included baseline forced expiratory volume in the first second, FVC, diffusion capacity for carbon monoxide (DLco), the Composite Physiological Index (CPI) and longitudinal FVC measurements. The start and end FVC measurements considered in the longitudinal analyses were within 3 months of the respective CT scan dates.

### Individual visual CT analysis

All baseline CTs were evaluated by a specialist thoracic radiologist (JJ) with 12 years of imaging experience and were classified according to the 2018 American Thoracic Society/European Respiratory Society/Latin American Thoracic Society/Japanese Respiratory Society international consensus guidelines.^7^ Each CT in cohort 1 was scored independently by two radiologists (GC and JB) with 3 and 4 years of thoracic imaging experience, respectively. Each CT in cohort 2 was scored independently by two radiologists (BG and AJP) with 3 and 5 years of thoracic imaging experience, respectively. Observers were blinded to all clinical information and the time points of the serial CTs. CT analysis involved interrogating images on dual-monitor workstations. CT patterns were classified according to the Fleischer Society glossary of terms^8^ with the following modifications: areas of increased density lung with overlying reticulation or traction bronchiectasis were characterised as ground glass. Increased density lung with no overlying reticulation, representing pure ground-glass opacity, was not quantified as it was felt likely to represent inflammation rather than interstitial fibrosis.

**Figure 1:**
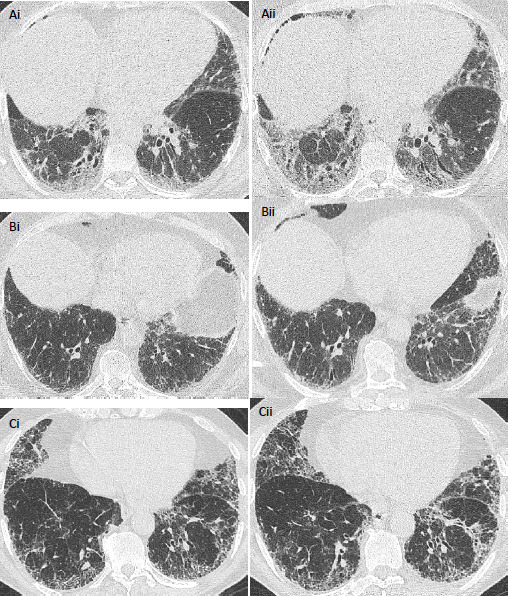
Serial axial CT images in patients with idiopathic pulmonary fibrosis. In a 50-year-old male patient who did not receive antifibrotic medication and who demonstrated a >10% annualised FVC decline, images acquired 6 months apart (Ai, ii) show change in traction bronchiectasis categorised as markedly worsened (score=5) by scoring radiologists. In a 62-year-old male patient who received antifibrotic medication (Bi, ii), images acquired 13 months apart show annualised FVC decline between 5.0% and 9.9%, and change in traction bronchiectasis was categorised as mildly worsened (score=4). In a 77-year-old man who did not receive antifibrotic medication (Ci, ii) and who had CTs acquired 15 months apart, change in traction bronchiectasis severity (Score=3) and annualised FVC decline (−5.0% to 4.9%) were both considered stable. Parenchymal changes visible on the CT may reflect disease maturation rather than disease progression. FVC, forced vital capacity.

Change in total ILD extent and total lung change in extents of ground-glass opacity, reticular pattern and honeycombing and severity of traction bronchiectasis (figure 1) were all scored on a categorical 5-point scale: 1=markedly improved, 2=slightly improved, 3=no change, 4=slightly worsened and 5=markedly worsened. CT pairs also had total ILD extent change scored on a lobar basis across six lobes (with the left middle lobe demarcated by the origin of the lingula bronchus). Results for lobar scores of ILD extent change are included in the online supplementary appendix. Consensus formulation for visual scores used the continuous learning method,^9^ whereby each CT was consensed by the scorers immediately after each CT read. CT change scores were adjusted to reflect the true timepoints of the CT pairs prior to statistical analysis. Accordingly, in a case where chronological randomisation meant that the second timepoint CT had been examined under the assumption that it was the first CT, and the first CT was assumed to be the second timepoint CT, observer scores of disease improvement were changed to equivalent scores of disease worsening. For example, a score of 1 or 2 was converted to a score of 5 or 4, respectively.

10.1136/thoraxjnl-2019-213865.supp1 Supplementary data

### Statistical analysis

Data are given as medians or means with SD, or numbers of patients with percentages where appropriate. Interobserver variation for visual scores was assessed using the quadratic-weighted kappa statistic for categorical variables. Weighted kappa coefficients were categorised as follows: poor (0–0.20), fair (0.21–0.40), moderate (0.41–0.60), good (0.61–0.80) and excellent (0.81–1.00). Student’s t-test was used to measure mean differences between continuous variables, and the χ^2^ test evaluated differences between categorical variables.

For both cohorts, the temporal trajectory of subjects’ FVC volumes was modelled using a linear mixed effect (LME) model with fixed effects of baseline age, sex (male/female), baseline antifibrotic use (never/ever) and study time, along with random intercepts and random slopes. We then estimated the change in FVC volume from baseline to follow-up CT measurement times by taking the difference in the LME models’ predicted FVC volume at these times.

In each cohort individually and in the combined population, we performed a survival analysis for each of the 11 CT variables of interest using a separate multivariate Cox regression. Time was measured from the second CT, and an event was either death or transplantation. We modelled survival as a function of the change in the CT variable with adjustment for the estimated change in FVC volume (estimated via the LME model), baseline age, sex, baseline DLco and baseline antifibrotic use. We adjusted the p values of the CT variables’ HRs for multiple comparisons by calculating the effective number of independent tests via the method of Li and Ji.^10^ In four study patients where baseline DLco values were unavailable, the mean population average DLco was imputed.

In the combined population, we also examined whether CT-derived change scores predicted mortality in three groups of subjects: those with an annualised FVC volume decline of at least 10% (definite FVC decline), those with annualised decline between 5% and <10% (marginal FVC decline), and those with annualised decline between −5% and <5% (FVC stability). We first estimated FVC volume trajectories for all 211 subjects using LME modelling with the same fixed and random effects as before. We estimated the change in FVC volume over the CT scanning period, as well as the annualised FVC volume decline over this period. We then performed separate survival analyses for each CT variable for the 107 subjects with an annualised FVC volume decline of at least 10%, the 53 subjects with decline between 5% and <10%, and the 36 subjects with decline between 0% and <5%, with multiple comparisons correction as before. Logistic regression analyses were used to identify relationships between a usual interstitial pneumonia (UIP) pattern on baseline CT and annualised FVC decline thresholds. A p value threshold of ≤0.05 was considered significant.

## Results

### Baseline analyses

Cohort 1 comprised 103 patients with IPF presenting to the Royal Brompton Hospital, London. Cohort 2 comprised 108 patients: St. Antonius Hospital, Utrecht (n=52), Ege Hospital Izmir, Turkey (n=46), and Southampton General Hospital, UK (n=10). Eight patients in the St Antonius population who underwent a lung transplant were censored at the date of transplantation. No patients were lost to follow-up.

Patient age, gender and baseline lung function tests were similar between both cohorts (table 1). Significantly more patients in cohort 2 received antifibrotic medication than those in cohort 1. A definite UIP pattern on CT was significantly more common in cohort 1 than cohort 2, which was also reflected in the increased mortality seen in cohort 1 (table 1). Patients in cohort 2 also had a slightly shorter interval between CTs.

**Table 1:** Patient age, gender and baseline values for various pulmonary function indices in two cohorts of patients with idiopathic pulmonary fibrosis

Variable	Cohort 1 (n=103)	Cohort 2 (n=108)
Median age (years)	67 (60–73)	65 (58–71)
Male/female (numbers)	85/18	80/28
Alive/dead (numbers)	33/70	52/56*
Mean CT time interval (years)	1.1±0.4	1.0±0.4*
Antifibrotic use (Y/N)	33/70	74/34**
Definite UIP pattern on CT (Y/N)	45/58	32/76*
Baseline FVC (% predicted)	74.3±20.0	75.9±19.6
Baseline DLco (% predicted)	41.3±12.2	48.2±14.4
Baseline CPI (% predicted)	50.6±11.4	46.2±12.3

Data represent mean values with SDs or medians with IQRs unless otherwise indicated.

*P<0.05, **P<0.001.

CPI, Composite Physiological Index; DLco, diffusion capacity for carbon monoxide; FVC, forced vital capacity; N, no; UIP, usual interstitial pneumonia; Y, yes.

The utility of any visual CT scoring system depends on its consistency in interpretation between observers. Therefore, we specifically examined two distinct IPF populations (cohort 1 was single centred with homogenous CT acquisitions, while cohort two was international with variable CT acquisitions) scored by two independent scorer pairs. We intentionally selected scorer pairs that were not experienced subspecialists, to better reflect real-world results from using the simple categorical scores of change. Agreement between observers for change in CT variables was weakest for honeycombing across both cohorts. However, interobserver agreement was good/excellent for all variables (table 2 and online supplementary table 1).

**Table 2:** Weighted kappa measurements indicating variation in visual scores of disease change on CT between the pairs of radiologist scorers

Visual CT variable	Cohort 1	Cohort 2
ILD change	0.87	0.87
Ground-glass opacity change	0.79	0.65
Reticular pattern change	0.83	0.80
Honeycombing change	0.64	0.70
Traction bronchiectasis change	0.81	0.66

Separate pairs of scorers evaluated CTs of patients with idiopathic pulmonary fibrosis in each cohort.

ILD, interstitial lung disease.

### Longitudinal CT analyses

When cohort 1 and cohort 2 were separately examined using univariable Cox regression analysis (table 3 and online supplementary table 2), all CT variables of change except ground-glass opacity significantly predicted mortality. The same CT variables of change were the strongest predictors of mortality in both cohorts: honeycombing extent, traction bronchiectasis severity, right middle lobe and right and left lower lobe ILD extents. When all the global lung CT variables (total ILD extent, ground-glass opacity, reticulation, honeycombing and traction bronchiectasis) were examined together in a multivariable Cox regression model, change in ground-glass opacity and honeycombing independently predicted mortality in cohort 1, while change in traction bronchiectasis severity alone independently predicted mortality in cohort 2.

**Table 3:** Univariable Cox regression analysis in cohort 1 (n=103) and cohort 2 (n=108) demonstrating mortality prediction determined by change in various visual CT variables measured with 5-point ordinal scores

Study cohort	Visual CT variable change	HR	95% CI	P value
**Cohort 1**	Total ILD extent	1.89	1.30 to 2.73	0.001
	Ground-glass opacity extent	1.91	1.30 to 2.79	0.001
	Reticular pattern extent	1.54	1.07 to 2.22	0.02
	Honeycombing extent	2.21	1.50 to 3.28	0.00007
	Traction bronchiectasis severity	2.06	1.40 to 3.06	0.0003
**Cohort 2**	Total ILD extent	1.60	1.18 to 2.17	0.002
	Ground-glass opacity extent	1.40	1.00 to 1.97	0.054
	Reticular pattern extent	1.68	1.17 to 2.42	0.005
	Honeycombing extent	1.87	1.27 to 2.76	0.002
	Traction bronchiectasis severity	2.61	1.60 to 4.28	0.0001

ILD, interstitial lung disease.

### Longitudinal CT and PFT models

Change in traction bronchiectasis severity was the strongest independent CT predictor of mortality on multivariable Cox regression analyses adjusted for patient age, gender, antifibrotic use and baseline DLco when the two cohorts were examined separately. Following the combination of both cohorts, the Concordance Index for a Cox regression model evaluating patient age, gender, antifibrotic use and baseline DLco in the combined population was 0.65. When FVC decline was added to the model, the Concordance Index was 0.66. When CT measures of change were separately added to this model instead of FVC decline, change in traction bronchiectasis severity was the most powerful predictor of mortality: HR=2.14, 95% CI 1.59 to 2.88, p value=2.5×10^−6^, CI 0.70 (table 4 and online supplementary table 3). The results were maintained when baseline disease severity was evaluated using first CPI (online supplementary table 4) and then FVC. In a final mortality model that additionally incorporated FVC decline, change in traction bronchiectasis severity predicted mortality independent of the degree of FVC decline, again with adjustment for patient age, gender, antifibrotic use and baseline DLco (table 5). Correlations between FVC decline and traction bronchiectasis change for the study population were weak (n=211, R^2^=0.05, p=0.0006). Changes in lobar ILD scores were less powerful predictors of mortality than changes in traction bronchiectasis severity scores (online supplementary table 5).

**Table 4:** Multivariable Cox regression analyses models demonstrating mortality prediction determined by change in various visual CT variables (measured with 5-point ordinal scores) in the combined idiopathic pulmonary fibrosis cohorts (n=211)

Categorical change in visual CT variables	HR, 95% CI, P value, Concordance Index
Total ILD extent	1.68, 1.33 to 2.12, 5.9×10^–5^, 0.70
Ground-glass opacity extent	1.64, 1.28 to 2.09, 0.0004, 0.68
Reticular pattern extent	1.61, 1.26 to 2.05, 0.0007, 0.69
Honeycombing extent	1.90, 1.44 to 2.51, 2.7×10^–5^, 0.69
Traction bronchiectasis severity	2.14, 1.59 to 2.88, 2.5×10^–6^, 0.70

P values shown are adjusted for multiple comparisons.

Each visual CT variable was analysed in a separate model adjusted for patient age, gender, baseline disease severity using the diffusion capacity for carbon monoxide and antifibrotic use (never/ever).

ILD, interstitial lung disease.

**Table 5:** Multivariable Cox regression analysis models demonstrating mortality prediction determined by change in various visual CT variables (measured with 5-point ordinal scores) in the combined idiopathic pulmonary fibrosis population (n=211)

Categorical change in visual CT variables	Visual CT variable (HR, 95% CI, P value, Concordance Index)	FVC decline (HR, 95% CI, P value)
Total ILD extent	1.53, 1.19 to 1.97, 0.004, 0.68	0.55, 0.30 to 1.01, 0.053
Ground-glass opacity extent	1.49, 1.15 to 1.93, 0.01, 0.68	0.47, 0.27 to 0.82, 0.008
Reticular pattern extent	1.41, 1.07 to 1.86, 0.07, 0.68	0.53, 0.29 to 0.97, 0.04
Honeycombing extent	1.66, 1.22 to 2.26, 0.006, 0.68	0.52, 0.28 to 0.94, 0.03
Traction Bronchiectasis severity	1.95, 1.42 to 2.67, 0.0002, 0.69	0.52, 0.30 to 0.90, 0.02

P values shown are adjusted for multiple comparisons.

Each visual CT variable was analysed in a separate model adjusted for patient age, gender, baseline disease severity using the diffusion capacity for carbon monoxide, antifibrotic use (never/ever) and FVC decline calculated using mixed effects models.

FVC, forced vital capacity; ILD, interstitial lung disease.

### Examination of FVC decline thresholds

In the combined population, the ability with which change in visual CT scores could predict mortality was examined at varying thresholds of annualised FVC decline: ≥10% decline, n=107; 5.0%–9.9% decline, n=53; −5.0% to 4.9% change, n=47 (table 6 and online supplementary table 6). Patients with ≥10% FVC decline had significantly more severe disease at baseline (assessed using baseline DLco and CPI) than patients with <10% annualised FVC decline (n=104; four patients demonstrated an FVC change of >−5.0%). However, no significant difference in baseline disease severity (DLco and CPI) was seen between patients undergoing FVC declines of 5.0%–9.9% or ≥10%. The presence of a definite UIP pattern (vs a probable UIP pattern) at baseline did not distinguish between patients undergoing an annualised FVC decline of ≥10% or <10% and did not distinguish between patients undergoing an FVC decline of ≥10% from patients undergoing an FVC decline between 5.0% and 9.9%. Adjusting for the variable time interval between CT scans did not influence any of the mortality models. CT time interval was examined as a continuous variable (range 6–24 months) and as a 3-point categorical variable at 6 monthly intervals.

**Table 6:** Mortality outcome within subgroups of annualised FVC change for various visual CT variables

FVC decline range	Categorical change in visual CT variables	Visual CT variables (HR, 95% CI, P value, Concordance Index)	FVC decline (HR, 95% CI, P value)
FVC 5.0%–9.9% decline	Total ILD extent	3.09, 1.39 to 6.88, 0.03, 0.69	1.85, 0.04 to 78.77, 0.74
	Ground-glass opacity extent	2.36, 1.04 to 5.35, 0.20, 0.66	0.72, 0.02 to 29.67, 0.86
	Reticular pattern extent	3.06, 1.34 to 7.01, 0.04, 0.69	1.52, 0.04 to 54.22, 0.82
	Honeycombing extent	3.79, 1.54 to 9.36, 0.02, 0.71	0.81, 0.02 to 32.13, 0.91
	Traction bronchiectasis severity	5.20, 2.19 to 12.32, 0.0009, 0.71	1.33, 0.03 to 65.14, 0.89
FVC ≥10% decline	Total ILD extent	1.45, 1.06 to 1.99, 0.10, 0.57	0.97, 0.44 to 2.15, 0.94
	Ground-glass opacity extent	1.43, 1.04 to 1.96, 0.13, 0.57	0.89, 0.42 to 1.92, 0.78
	Reticular pattern extent	1.31, 0.93 to 1.84, 0.58, 0.56	0.98, 0.43 to 2.22, 0.96
	Honeycombing extent	1.39, 0.99 to 1.95, 0.28, 0.57	0.92, 0.42 to 2.03, 0.84
	Traction bronchiectasis severity	1.63, 1.12 to 2.36, 0.049, 0.59	0.97, 0.445 to 2.10, 0.94

P values shown are adjusted for multiple comparisons.

The visual CT variables (measured with 5-point ordinal scores) were examined in separate multivariable Cox regression models adjusted for patient age, gender, baseline disease severity (using diffusion capacity for carbon monoxide) and antifibrotic use (never/ever). Analyses were performed in patients with an annualised FVC decline of 5.0%–9.9% (n=53) and ≥10% (n=107).

FVC, forced vital capacity; ILD, interstitial lung disease.

In the patient group with the largest FVC decline (≥10%), change in traction bronchiectasis severity independently predicted mortality (table 6). When examined in models containing visual CT variables, FVC declines of ≥10%, assessed as continuous variables, did not independently predict survival in any of the models (table 6). No CT variables were linked to mortality prediction in patients with the smallest FVC declines (−5.0 to 4.9%), and in this group of patients, no individual had any change in honeycombing identified by either scorer pair.

**Figure 2:**
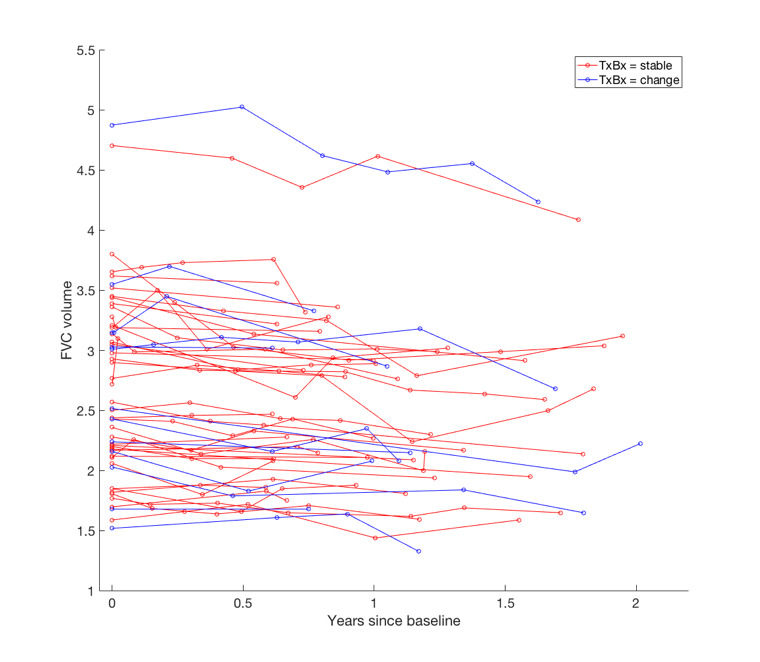
Spaghetti plot demonstrating longitudinal change in FVC from the time of the baseline CT scan. Patients have been classified as having no change in traction bronchiectasis (red) and change in traction bronchiectasis (blue). The start and end FVC measurements considered in the longitudinal analyses were within 3 months of the respective CT scan dates. FVC, forced vital capacity; TxBx, traction bronchiectasis.

In patients with marginal FVC declines of 5.0%–9.9%, change in traction bronchiectasis severity was the strongest independent predictor of mortality when examined against FVC decline measured as a continuous variable (table 6). No significant correlations were identified between FVC decline and traction bronchiectasis change in patients with marginal FVC declines. In models containing visual CT variables, when FVC declines of 5.0%–9.9% were assessed as continuous variables, FVC measures did not independently predict survival in any of the models, echoing results in patients with IPF with an FVC decline of ≥10%. Five of 53 (9%) patients with an FVC decline between 5.0% and 9.9% had honeycombing change identified on CT pairs. However, 12/53 (23%) patients with an FVC decline between 5.0% and 9.9% were identified as having a change in traction bronchiectasis severity (figure 2).

## Discussion

Our study specifically set out to examine whether change in visual CT variables across serial CT studies in patients with IPF could be used to resolve annualised marginal FVC declines of between 5.0% and 9.9% predicted. Marginal FVC changes are challenging to interpret in routine clinical practice as they could reflect either measurement noise or genuine physiological deterioration. We demonstrate that change in traction bronchiectasis severity, scored using a simple 5-point categorical scale, predicts mortality independent of patient treatment with antifibrotics and baseline disease severity. Importantly, in patients with FVC declines between 5.0% and 9.9%, traction bronchiectasis severity change identified more patients with disease progression than honeycombing extent change and could therefore be used to determine whether the functional decline is clinically meaningful.

Several previous baseline studies in patients with IPF^11 12^ and other idiopathic and non-idiopathic fibrosing lung diseases^13–18^ have underlined the prognostic value of visual estimations of traction bronchiectasis severity using categorical scores. Yet, no previous studies have examined whether change in visual traction bronchiectasis scores can predict mortality in patients with IPF. Prior baseline studies examining traction bronchiectasis have highlighted its improved interobserver agreement when compared with scores of parenchymal pattern extents such as honeycombing. Our study, though examining longitudinal change on CTs, has demonstrated good-to-excellent agreement between observer pairs for traction bronchiectasis change, with agreement better than that seen for honeycombing change.

Change in traction bronchiectasis severity demonstrated the strongest prognostic signal in both patient cohorts as well as in patients with indeterminate FVC declines. As awareness around the prognostic value of longitudinal CT analysis grows by the year, patients with IPF are likely to undergo more frequent CT imaging necessitating a better understanding of longitudinal CT measures of deterioration. Change in traction bronchiectasis severity predicted mortality independent of patient therapy, suggesting that it could represent an important measure of disease worsening in drug trials. Twenty-three per cent of patients with marginal FVC declines were identified as exhibiting change in traction bronchiectasis severity. A sensitive measure of disease worsening will have particular importance in non-inferiority IPF trials where standard of care with antifibrotic therapy will result in a high proportion of patients undergoing marginal FVC declines. Reassuringly, as well as strongly predicting survival in a homogenous single-centre study cohort, change in traction bronchiectasis severity strongly predicted outcome in cohort 2, where the multicentred nature of the CT imaging analysed better reflects the range of image acquisitions captured in drug trials. Our positive findings suggest that potentially noisy and heterogenous imaging data can still prove prognostically valuable.

Our findings also have relevance in other fibrosing lung diseases. With elucidation of the progressive fibrotic phenotype,^19^ it is now acknowledged that identifying disease worsening over time may be just as important as reaching a specific diagnosis. Patients enrolled in the INBUILD trial,^3^ examining non-IPF progressive fibrotic conditions, are likely to undergo FVC declines of smaller magnitudes than those typically seen in patients with IPF. The INBUILD trial protocol required patients without IPF undergoing FVC declines between 5.0% and 9.9% to demonstrate symptomatic worsening or worsening on CT imaging.^3^ Our findings demonstrate that progression on CT imaging can act as a surrogate for mortality in patients whose FVC declines when taken alone may not confidently suggest progression. Importantly, the mortality linkages identified using our CT variables indicate that they reflect disease progression (involvement of new lung tissue) rather than disease maturation (evolving changes in lung tissue already damaged). We also demonstrate that rather than evaluating total ILD extent progression as has been traditionally used, change in traction bronchiectasis might be the best of the existing suite of CT variables to examine.

A major focus of CT image analysis in recent years has involved using computer tools to quantify lung damage, both at baseline and longitudinally. Volumetric computer analysis has the potential to afford a greater degree of precision and sensitivity for detecting lung damage when compared with more crude semiquantitative visual CT scores. Yet, several challenges may constrain the uptake of computer technologies. Analytical tools employed on longitudinal CT imaging will need to take into account the noise associated with variations in CT acquisition parameters at baseline and between longitudinal scan pairs, as well as variation in patient respiratory effort across CT timepoints. Volumetric quantitation of lung damage evolution, as performed by computer tools, can also underestimate increases in the extent of damage in a disease such as IPF, where affected lung shrinks in volume and the spared lung hyperexpands in compensation. Over time, though ILD extent increases, a proportion of the involved lung shrinks, resulting in underestimation of disease worsening over time. Lastly, computer tools are often proprietary and may consider unique variables that cannot be quantified by the human eye, making comparisons between clinicians, hospitals or countries challenging.

In contrast, longitudinal visual CT analysis is free and, to date, has examined well-accepted CT variables that have been long defined by radiology glossaries.^8^ While variation resulting from patient-related respiratory effort will still account for some measurement noise, careful selection of appropriate visual CT variables may negate problems with lung shrinkage that could underestimate disease progression. For example, traction bronchiectasis and honeycombing extent scores constitute variables that represent larger proportions of damaged lung as disease worsens. Their linear relationship with disease progression may account for their sensitivity when compared with more traditionally examined global scores of worsening, such as total ILD extent.

There were several limitations to the current study. As the study was retrospective, CT imaging was not performed at predefined regular intervals. Yet, the mean interval between CTs was approximately 1 year in both cohorts, which is within the bounds of follow-up duration expected in IPF drug trials. Different scorer pairs evaluated the two cohorts and showed differences in agreement across several parenchymal patterns. Our a priori aim had been to examine real-world observer interpretations of CT images, using real-world clinical methods (side-by-side longitudinal CT examination) and not relying on adjudication by experienced subspecialist radiologists. Accordingly, we believe our methods reflect a realistic interpretation of longitudinal CT images.

In conclusion, our study has shown that change in traction bronchiectasis is a reproducible measure of disease progression in IPF. Change in traction bronchiectasis can be used to resolve indeterminate FVC declines, which are likely to be seen with increasing frequency in patients with IPF receiving antifibrotics, and potentially in patients without IPF with a progressive fibrotic phenotype.
