# Supplementary material for: Serial CT analysis in idiopathic pulmonary fibrosis: comparison of visual features that determine patient outcome
Source: Thorax. 2020 Apr 28;75(8):648–54. doi: 10.1136/thoraxjnl-2019-213865 (PMC7402558; doi:10.1136/thoraxjnl-2019-213865)
Supplement: Supplementary data [file thoraxjnl-2019-213865supp001.pdf]

Supplementary Appendices

**Visual CT Variable**	**Cohort 1**	**Cohort 2**
Right upper lobe ILD extent change	0.87	0.83
Left upper lobe ILD extent change	0.83	0.77
Right middle lobe ILD extent change	0.87	0.71
Left middle lobe ILD extent change	0.83	0.75
Right lower lobe ILD extent change	0.83	0.77
Left lower lobe ILD extent change	0.83	0.80

Supplementary Table 1. Weighted Kappa measurements indicating variation in visual scores of disease change on CT between the pairs of radiologist scorers. Separate pairs of scorers evaluated CTs of idiopathic pulmonary fibrosis patients in Cohort 1 and 2. ILD =interstitial lung disease.

Study Cohort	Visual CT variable change	Hazard Ratio	95% Confidence Interval	P value
**Cohort 1**	Right upper lobe ILD extent	1.69	1.20-2.40	0.003
	Left upper lobe ILD extent	1.72	1.24-2.39	0.001
	Right middle lobe ILD extent	2.02	1.40-2.92	0.0002
	Left middle lobe ILD extent	1.60	1.14-2.23	0.006
	Right lower lobe ILD extent	1.99	1.41-2.82	0.0001
	Left lower lobe ILD extent	1.90	1.37-2.64	0.0001
**Cohort 2**	Right upper lobe ILD extent	1.52	1.03-2.24	0.04
	Left upper lobe ILD extent	1.50	1.03-2.18	0.03
	Right middle lobe ILD extent	1.98	1.37-2.84	0.0002
	Left middle lobe ILD extent	1.49	1.03-2.16	0.04
	Right lower lobe ILD extent	1.56	1.14-2.14	0.006
	Left lower lobe ILD extent	1.58	1.15-2.16	0.004

Supplementary Table 2. Univariable Cox regression analysis in Cohort 1 (n=103) and Cohort 2 (n=108) demonstrating mortality prediction determined by change in various visual CT variables measured with 5-point ordinal scores. ILD=interstitial lung disease.

**Categorical change in visual CT variables**	**HR, 95% CI, p value, Concordance Index**
Right upper lobe ILD extent	1.56, 1.22-2.16, 0.002, 0.68
Left upper lobe ILD extent	1.56, 1.23-1.98, 0.0001, 0.68
Right middle lobe ILD extent	1.86, 1.43-2.41, 0.00001, 0.70
Left middle lobe ILD extent	1.53, 1.20-1.94, 0.002, 0.68
Right lower lobe ILD extent	1.67, 1.34-2.09, 0.00003, 0.70
Left lower lobe ILD extent	1.73, 1.39-2.16, 5.1×10^{-6}, 0.70

Supplementary Table 3. Multivariable Cox regression analyses models demonstrating mortality prediction determined by change in various visual CT variables (measured with 5-point ordinal scores) in the combined population of idiopathic pulmonary fibrosis patients (n-211). Each visual CT variable was analysed in a separate model adjusted for patient age, gender, baseline disease severity using the diffusion capacity for carbon monoxide and antifibrotic use (never/ever). HR=Hazard ratio, CI=confidence Interval, ILD=interstitial lung disease. P values shown are adjusted for multiple comparisons.

Categorical change in visual CT variable	HR, 95% CI, p value, Concordance Index)
Total ILD extent	1.67, 1.33-2.09, $4.7x10^{-5}$, 0.70
Ground glass opacity extent	1.62, 1.27-2.06, 0.0005, 0.69
Reticular pattern extent	1.62, 1.28-2.07, 0.0004, 0.70
Honeycombing extent	1.90, 1.44-2.51, $2.5x10^{-5}$, 0.70
Traction Bronchiectasis severity	2.12, 1.58-2.84, $2.7x1\text{-}^{-6}$, 0.71
Right upper lobe ILD extent	1.54, 1.20-1.97, 0.003, 0.68
Left upper lobe ILD extent	1.54, 1.22-1.94, 0.003, 0.69
Right middle lobe ILD extent	1.84, 1.42-2.38, $1.5x10^{-5}$, 0.71
Left middle lobe ILD extent	1.51, 1.19-1.91, 0.003, 0.69
Right lower lobe ILD extent	1.65, 1.32-2.05, $3.8x10^{-5}$, 0.71
Left lower lobe ILD extent	1.77, 1.42-2.20, $1.7x10^{-6}$, 0.71

Supplementary Table 4. Multivariable Cox regression analyses models demonstrating mortality prediction determined by change in various visual CT variables (measured with 5-point ordinal scores) in the combined population of idiopathic pulmonary fibrosis patients (n=211). Each visual CT variable was analysed in a separate model adjusted for patient age, gender, baseline disease severity using the composite physiologic index and antifibrotic use (never/ever). HR=Hazard ratio, CI=confidence interval, ILD=interstitial lung disease.

**Categorical change in visual CT variables**	**Visual CT variable (HR, 95% CI, p value, Concordance Index)**	**FVC decline (HR, 95% CI, p value)**
Right upper lobe ILD extent	1.32, 0.99-1.75, 0.29, 0.66	0.49, 0.26-0.89, 0.02
Left upper lobe ILD extent	1.33, 1.02-1.74, 0.18, 0.67	0.49, 0.27-0.91, 0.02
Right middle lobe ILD extent	1.67, 1.25-2.21, 0.002, 0.69	0.55, 0.30-1.01, 0.05
Left middle lobe ILD extent	1.32, 1.01-1.73, 0.23, 0.67	0.49, 0.26-0.92, 0.03
Right lower lobe ILD extent	1.52, 1.20-1.94, 0.003, 0.68	0.55, 0.30-1.02, 0.06
Left lower lobe ILD extent	1.60, 1.24-2.06, 0.001, 0.69	0.64, 0.34-1.22, 0.18

Supplementary Table 5. Multivariable Cox regression analyses models demonstrating mortality prediction determined by change in various visual CT variables (measured with 5-point ordinal scores) in the combined idiopathic pulmonary fibrosis population (n=211). Each visual CT variable was analysed in a separate model adjusted for patient age, gender, baseline disease severity using the diffusion capacity for carbon monoxide, antifibrotic use (never/ever) and forced vital capacity decline calculated using mixed effects models. HR=Hazard ratio, CI=confidence interval, ILD=interstitial lung disease. P values shown are adjusted for multiple comparisons.

FVC decline range	Categorical change in visual CT variables	Visual CT variables (HR, 95% CI, p value, Concordance Index)	FVC decline (HR, 95% CI, p value)
**FVC 5.0-9.9% decline**	Right upper lobe ILD extent	3.56, 1.55-8.14, 0.01, 0.68	3.88, 0.05-319.56, 0.55
	Left upper lobe ILD extent	4.03, 1.72-9.47, 0.007, 0.73	4.01, 0.05-311.73, 0.53
	Right middle lobe ILD extent	3.18, 1.44-7.02, 0.02, 0.69	0.56, 0.01-38.57, 0.77
	Left middle lobe ILD extent	4.14, 1.92-8.93, 0.001, 0.72	0.94, 0.01-59.60, 0.98
	Right lower lobe ILD extent	2.34, 1.04-5.27, 0.20, 0.65	0.94, 0.02-40.92, 0.98
	Left lower lobe ILD extent	2.06, 0.96-4.38, 0.31, 0.65	0.71, 0.02-30.35, 0.86
**FVC ≥10% decline**	Right upper lobe ILD extent	1.13, 0.81-1.58, 1.00, 0.55	0.86, 0.38-1.98, 0.73
	Left upper lobe ILD extent	1.15. 0.84-1.59, 1.00, 0.55	0.87, 0.38-1.95, 0.73
	Right middle lobe ILD extent	1.52, 1.09-2.11, 0.07, 0.58	0.99, 0.44-2.22, 0.99
	Left middle lobe ILD extent	1.11, 0.81-1.51, 1.00, 0.54	0.84, 0.37-1.88, 0.67
	Right lower lobe ILD extent	1.40, 1.05-1.86, 0.10, 0.58	0.92, 0.42-2.03, 0.84
	Left lower lobe ILD extent	1.54, 1.15-2.08, 0.02, 0.60	1.13, 0.50-2.56, 0.76

Supplementary Table 6. Various visual CT variables (measured with 5-point ordinal scores) were examined in separate multivariable Cox regression models adjusted for patient age, gender, baseline disease severity (using diffusion capacity for carbon monoxide) and antifibrotic use (never/ever). Analyses were performed in patients with an annualised forced vital capacity decline of 5.0-9.9% (n=53) and ≥10% (n=107). HR=Hazard ratio, CI= confidence interval, ILD=interstitial lung disease. P values shown are adjusted for multiple comparisons.
